# Caffeine treatment for apnea in infants and children: protocol for a systematic review and meta-analysis

**DOI:** 10.1186/s13643-026-03222-w

**Published:** 2026-06-08

**Authors:** Michelle Yu, Hana Sharifi, Sofia Quon, Kaitryn Campbell, Bram Rochwerg, David J. Zorko, Melissa J. Parker

**Affiliations:** 1 Michael G. DeGroote School of Medicine, McMaster University, Hamilton, Canada; 2 Bachelor of Arts and Sciences Program, McMaster University, Hamilton, Canada; 3 Campbell Information Consulting, Hamilton, Ontario Canada; 4 Department of Medicine, McMaster University, DBRI, Rm C5-106 and 107, 237 Barton Street East, Hamilton, Ontario L8L 2X2 Canada; 5 Department Health Research Methods, Evidence and Impact, McMaster University, 1200 Main St W., Hamilton, Ontario L8N 3Z5 Canada; 6 Division of Pediatric Critical Care, Department of Pediatrics, McMaster Children’s Hospital and McMaster University, 1280 Main St. W. HSC 3E-20, Hamilton, Ontario L8S 4K1 Canada; 7 Division of Emergency Medicine, Department of Pediatrics, The Hospital for Sick Children, and University of Toronto, 555 University Avenue, Toronto, Ontario M5G 1X8 Canada

**Keywords:** Caffeine, Apnea, Pediatrics, Emergency treatment, Critical care

## Abstract

**Background:**

Caffeine citrate is recommended to treat apnea of prematurity in premature neonates based on high-quality evidence demonstrating improved outcomes. In contrast, the prescription of caffeine for mature neonates and infants hospitalized with apnea is based on a paucity of evidence. We present here our protocol for a systematic review to synthesize existing data on the safety and effectiveness of caffeine treatment for apnea in hospitalized pediatric patients, with findings expected to focus on mature neonates and infants.

**Methods:**

We developed this systematic review protocol in accordance with the PRISMA guidelines and PRISMA-P checklist. The search strategy was developed with the assistance of an information specialist. Information sources will include MEDLINE, Embase, CINAHL, and CENTRAL databases, as well as unpublished literature sources. Eligible studies will include randomized controlled trials, non-randomized trials, and observational studies investigating caffeine (of any formulation, administered by any route, of any duration) versus placebo, usual care, or non-methylxanthine comparator. The population of interest is children aged 0–17 years with apnea presenting to emergency, critical care, or in-patient settings; studies of premature babies receiving treatment for apnea of prematurity will be excluded. Critical outcomes include total duration of respiratory support, ventilator-free days, duration of invasive mechanical ventilation, duration of non-invasive respiratory support, time to apnea cessation, and serious adverse events. Two authors will independently screen identified studies against prespecified eligibility criteria. Data from included studies will be abstracted in duplicate and entered into our data abstraction form. Risk of bias for each included study will be independently assessed by two reviewers using the appropriate risk of bias tool. If appropriate, we will perform a meta-analysis. The quality of evidence will be assessed using the Grading of Recommendations Assessment, Development and Evaluation (GRADE) methodology, with results presented in a summary of findings table.

**Discussion:**

We will undertake the first rigorous evidence synthesis examining the safety and efficacy of caffeine treatment for apnea in hospitalized pediatric patients, where studies of premature babies with apnea of prematurity will be specifically excluded. We aim to fill the identified knowledge gap with this systematic review.

**Systematic review registration:**

PROSPERO CRD420251146484.

**Supplementary Information:**

The online version contains supplementary material available at 10.1186/s13643-026-03222-w.

## Rationale

Caffeine citrate is a well-established treatment for apnea of prematurity (AoP) in infants born at less than 35 weeks of gestation [1]. AoP is defined as a complete cessation of breathing in premature infants that lasts more than 15 s, in addition to hypoxia or bradycardia [1]. The development of AoP is attributed to an immature and thus depressed central respiratory drive and suppressed peripheral chemoreceptors, which are necessary for supporting upper airway muscle tone and activating carotid bodies to stimulate the respiratory system [2]. Much of the apnea observed at this stage is idiopathic, developing after the first 24 to 72 h of life, and is common [2]. Recurrent episodes of AoP can be dangerous and have been associated with later development of bronchopulmonary dysplasia or neurodevelopmental impairment (Bayley Mental Developmental Index or Psychomotor Developmental Index < 70, cerebral palsy, or blindness) [3]. High-level randomized controlled trial evidence has demonstrated the effectiveness of caffeine to treat AoP through a clear pharmacological mechanism of action [1]. Due to its proven effectiveness, caffeine treatment of AoP in premature neonates became the standard of care following publication of the CAP trial [4]. Beyond its use in premature neonates, however, caffeine prescription for the treatment of apnea in other pediatric populations does not appear to be supported by high-level evidence.

Apnea, a clinical problem with different potential causes, is also a significant medical issue in non-premature (i.e., ≥ 37 weeks GA) neonates, infants, and older children. A broad differential diagnosis must be considered in infants presenting with apnea including, viral infections, bacterial sepsis with either direct (e.g., meningitis) or remote (e.g., pneumonia, urinary tract infections) effects on the central nervous system (CNS), other CNS pathophysiologic disorders such as seizures, or physiologic events related to position or feeding.[5]. There are a number of infections which can cause central apnea in infants and neonates [6]. The classic pathogen is Respiratory Syncytial Virus (RSV); however, other viruses have been implicated, including Adenovirus, Enteroviridae, Influenza A, Parainfluenza 3, Parainfluenza 4, and Rhinovirus [5]. The pathophysiology and mechanisms that induce new onset apnea during the course of illness with such viral infections remains unclear. Bacterial pathogens can also cause apnea through the common pathway of sepsis [7]. Bordatella Pertussis characteristically presents with apnea in young infants [8, 9].

Despite caffeine treatment being well-established for treating AoP, little is known about whether it is similarly safe and effective for treatment of apnea in mature neonates and children. The pathophysiology of apnea in this population may be due to different mechanisms and pathways that do not necessarily respond to treatment with caffeine. Some physicians are prescribing caffeine for this non-preterm population presenting with apnea, in an effort to avoid escalation of respiratory support including invasive mechanical ventilation [10]. The desire to avoid invasive therapies and the need for critical care admission are laudable clinical goals. One single-center study revealed that 40% of children who required invasive mechanical ventilation for ≥12 h in a pediatric critical care unit experienced a mechanical ventilation-related adverse event [11]. Children requiring Pediatric Intensive Care Unit (PICU) management are also at risk for Post-Intensive Care Syndrome (PICS-p), where the latter negatively impacts recovery trajectory and health-related quality of life [12]. Nonetheless, treatment decisions should be evidence-based and extrapolation of evidence from premature babies treated for apnea of prematurity to mature babies and infants with apnea may be inappropriate. The prescription of caffeine citrate to pediatric patients hospitalized with apnea appears to be based on a paucity of evidence, providing rationale for this study.

## Objectives

1. To conduct a systematic review to identify and summarize published research on the safety and effectiveness of caffeine compared to non-methylxanthine comparator, placebo, or usual care in the treatment of apnea in children 0–17 years of age.
2. To conduct a meta-analysis of identified studies if methodologically appropriate.
3. To conduct subgroup analyses for subgroups of interest.

## Methods

We developed this protocol in accordance with the Preferred Reporting Items for Systematic Review and Meta-Analysis Protocols (PRISMA-P) statement [13, 14]. A completed PRISMA-P checklist is provided (Appendix 1). The protocol has also been registered in the PROSPERO database (CRD420251146484).

### Eligibility criteria

#### Population

Children (i.e., 0–17 years of age) receiving emergency care, critical care, or in-patient treatment for apnea. We will include studies of mixed premature and mature babies and children provided the study focus is on management for an acute illness with episode(s) of apnea other than apnea of prematurity. We will exclude studies of premature babies with apnea of prematurity receiving treatment in the neonatal intensive care unit (NICU). NICU-based studies evaluating babies with acute onset apnea not characterized as AOP, will be included if at least 80% of the population are ≥ 37 weeks GA.

#### Intervention

The intervention of interest is caffeine of any formulation, prescribed at any dose, route (i.e., enteral or parenteral), frequency (i.e., bolus or infusion; single or multiple boluses), and for any treatment duration. We will include studies that administered caffeine for apnea at any time after presentation to hospital. We will not restrict studies based on whether or not they titrated the intervention dose or evaluated caffeine drug levels. We will include studies that administered caffeine for apnea treatment or prevention. We will exclude studies that evaluated non-caffeine methylxanthines for apnea treatment or prevention. We will exclude studies comparing caffeine treatment regimens that do not have a placebo or non-methylxanthine comparator group.

#### Comparator

Placebo, non-methylxanthine comparator, or usual care.

#### Outcomes

We will focus on evaluating critical and important outcomes related to duration and intensity of respiratory support, duration of apnea, length of PICU and hospital stay, and adverse events.

### Study designs to be included

We will include randomized controlled trials of any type including parallel, cluster-randomized, cross-over, and factorial designs. We will include but analyze separately non-randomized trials, prospective observational studies that include a comparator group, and retrospective observational studies that include a comparator group. Case reports, editorials, and letters or commentaries, as well as qualitative studies, will be excluded. Select data from unpublished literature will also be included, such as relevant conference proceedings, abstracts, theses, and dissertations. While we plan to include both randomized and non-randomized studies in our search, these study types will be separately pooled in the meta-analysis. For observational data we will not pool adjusted and non-adjusted analysis results.

The decision to include non-randomized studies in our systematic review is supported by GRADE guidance and informed by the limited number of randomized controlled trials assessing interventions in critically ill children [15, 16]. While including observational studies in systematic reviews can be important when no or a limited number of randomized controlled trials exist, this necessitates a stratified approach to data synthesis and certainty of evidence evaluation due to the expected lower quality of observational studies. Our planned approach is described further in the Data Synthesis and Certainty in Cumulative Evidence sections respectively.

### Study setting

Hospital emergency department or Hospital in-patient settings including Pediatric Intensive Care Unit (PICU) of any level including high-dependency/step-down care areas, Neonatal Intensive Care Unit (NICU) of any level, and other in-patient care areas, e.g., in-patient wards. We will exclude any studies focused on treatment in immediate postpartum care areas such as in the delivery room or adjacent resuscitation care areas focused on resuscitation and stabilization of babies immediately following birth.

### Timeframe

We will include studies of all follow-up durations.

### Report characteristics

We will include studies from any year, of any publication status (published or unpublished), available in any language, which fulfill our design criteria.

## Information sources

### Electronic sources

We designed our search strategy with the advice and assistance of two independent health information specialists with expertise in systematic review methods. The search strategy was piloted using 3 seed articles to ensure capture of known articles meeting our eligibility criteria. While apnea is a clinically heterogeneous problem, in our experience caffeine treatment for apnea in pediatric patients is directed towards treatment of central rather than obstructive apnea. We opted not to exclude apnea subcategories, e.g., obstructive apnea, in our search strategy to avoid being overly restrictive.

The search strategy will be applied to the following databases from inception: Medical Literature Analysis and Retrieval System Online (MEDLINE), Excerpta Medica Database (Embase), Cumulated Index in Nursing and Allied Health Literature (CINAHL), and Cochrane Controlled Register of Trials (CENTRAL). We will include published conference abstracts meeting our eligibility criteria from the World Federation of Pediatric Intensive and Critical Care Societies (WFPICCS) Congress, the Society of Critical Care Medicine (SCCM) Critical Care Congress, the Pediatric Academic Societies (PAS) Congress, and the International Congress of the European Academy of Paediatrics (EAP) and the European Society for Paediatric Research (ESPR) from the past 3 years (2023–2025). To identify unpublished and ongoing clinical trials we will search ClinicalTrials.gov, ISRCTN: the UK’s Clinical Study Registry, and ICTRP: the World Health Organization’s International Clinical Trials Registry Platform. Unpublished literature will also be collected from PapersFirst, ProceedingsFirst, and ProQuest Dissertations and Theses Global. We will limit our search for unpublished literature data to the past 3 years.

### Other relevant sources

We will search the reference lists of studies identified for inclusion for potentially eligible publications not yet identified. Any such studies will be screened via the same process to determine if they are eligible for inclusion.

## Search strategy

The details of the finalized search strategy are provided in Table S1. The information specialist will execute the Medline, Embase, and CENTRAL searches simultaneously as a multi-file search within OVID, and the CINAHL search will be conducted separately via EBSCO. Search results will be exported as RIS files and uploaded into Covidence, followed by removal of duplicates within Covidence.

## Study records

### Data management

Following de-duplication, citations identified in the OVID multi-file search will be downloaded into EndNote and then imported to Covidence.

### Selection process

Citation screening will be conducted independently and in duplicate in two steps, including title and abstract screening (stage 1), followed by full-text screening (stage 2). The selection process will be completed using the Covidence platform (Veritas Health Innovation, Melbourne, Australia). During the first stage of screening by title and abstract, any citations considered potentially relevant by either reviewer will be moved on to the next stage. At stage 2, full-text screening will yield a further refined list of articles that fulfill our eligibility criteria. Any discrepancies between reviewers will be resolved by discussion and consensus. If necessary, where discrepancies cannot be resolved by consensus, we will employ adjudication by a third reviewer. A Preferred Reporting Items for Systematic Reviews and Meta-analyses flowchart illustrating the steps from study identification to inclusion, and reasons for any exclusions at stage 2, will be presented as Figure 1 in the results manuscript.

### Data collection process

Data collection will be accomplished through use of a data collection form. The data collection form will be created within Covidence and include items for recording study characteristics, population characteristics, intervention characteristics, control characteristics, and outcomes of interest. Two members of the study team experienced with data abstraction for systematic reviews will pilot the data abstraction form with at least two eligible studies, after which point the data collection form will be finalized.

Reviewers participating in formal data abstraction will receive training. Data extraction will be conducted independently (in duplicate) by two investigators. Data will be abstracted from the full-text publication, and where relevant from any referenced protocols, trial registries, or supplementary materials. Where necessary, data presented as medians will be converted to means as described within the statistical analysis section. Should only graphical results be provided for any pre-specified outcomes of interest, we will perform graphical data extraction using SourceForge Plot Digitizer (http://plotdigitizer.sourceforge.net) and have a second reviewer check this for accuracy. Reviewer discrepancies regarding abstracted data will be resolved by consensus, and if necessary, through review by a third investigator.

For any missing data or data that requires clarification, we will contact study authors up to 3 times in an attempt to rectify this. If issues related to missing or unclear data cannot be resolved, we will proceed with analysis of available data. The potential impact of any missing data on the results and risk of bias assessment will be reported.

## Outcomes and prioritization

The prioritized outcomes for our systematic review are presented in Table 1. For all critical and important outcomes, we will use the longest endpoint reported.

**Table 1:** Critical and important outcomes

**Critical outcomes**	**Data type; effect estimate**
1. Total/combined duration of all respiratory support (including invasive and non-invasive modes of support), (as defined by investigators)	Continuous; mean difference
2. Ventilator-free days (as defined by investigators)	Continuous; mean difference
3. Total duration of invasive mechanical ventilation (IMV)	Continuous; mean difference
4. Total duration of non-invasive respiratory support (as defined by investigators)	Continuous; mean difference
5. Time to apnea cessation (freedom from apneic events)	Continuous; mean difference
6. Serious adverse events	
a. Cardiac arrhythmia (SVT, VT, VF, or other malignant arrhythmia) requiring treatment/intervention	Categorical; risk or odds ratio
b. Sinus tachycardia of sufficient concern to prompt downtitration/pause/or discontinuation of caffeine	Categorical; risk or odds ratio
c. Seizure(s) (as defined by investigators)	Categorical; risk or odds ratio
d. Death	Categorical; risk or odds ratio
e. Other reported SAE or SUADR attributed to caffeine	Categorical; risk or odds ratio
**Important outcomes**	
1. Type of respiratory support type received	
a. Invasive mechanical ventilation	Categorical; risk or odds ratio
b. Biphasic positive airway pressure (BIPAP)	Categorical; risk or odds ratio
c. Continuous positive airway pressure (CPAP)	Categorical; risk or odds ratio
d. High flow nasal cannula (HFNC)	Categorical; risk or odds ratio
2. Duration of non-invasive respiratory support types	
a. Biphasic positive airway pressure (BIPAP)	Continuous; mean difference
b. Continuous positive airway pressure (CPAP)	Continuous; mean difference
c. High flow nasal cannula (HFNC)	Continuous; mean difference
3. Apnea burden (as defined by investigators)	Measure dependent
4. Length of PICU (or other critical care area) stay	Continuous; mean difference
5. Length of hospital stay (as defined by investigators)	Continuous; mean difference
6. Long term outcomes	
a. Neurodevelopmental delay or impairment (as defined by investigators) ascertained after hospital discharge	Measure dependent
b. Any long term outcomes measured ≥30 days following cessation of caffeine/comparator treatment	Measure dependent
7. Adverse events not meeting SAE or SUADR criteria	
a. Feeding Intolerance (as defined by investigators)	Categorical; risk or odds ratio
b. Other	Categorical; risk or odds ratio

*PICU *Pediatric Intensive Care Unit, *SAE *serious adverse event, *SUADR *serious unexpected adverse drug reaction, *SVT *supraventricular tachycardia, *VF *ventricular fibrillation, *VT *ventricular tachycardia

Pooled effect estimates will be reported with 95% confidence intervals. Risk or odds ratios will be reported for categorical outcomes and mean differences for continuous outcomes, with RCT pooled results and non-randomized pooled results reported separately.

## Risk of bias in individual studies

Two reviewers will independently and in duplicate assess each included study for risk of bias, using the risk of bias tool appropriate to the study design. Each tool assesses the risk of bias of a study across multiple domains, followed by an overall judgement regarding the risk of bias of a study. For randomized controlled trials, the Cochrane risk-of-bias tool (ROB-2) will be used [17]. RCTs will be assigned to one of three risk of bias categories: (1) low, (2) high, or (3) unclear. For non-randomized studies, the ROBINS-I tool (Risk Of Bias In Non-randomized Studies of Interventions), Version 2 (2024) will be used [18]. Non-randomized studies will be assigned to one of four risk of bias categories: (1) low, (2) moderate, (3) serious, or (4) critical. Any discrepancies in risk of bias assessment for a study will be resolved through discussion or adjudicated by a third reviewer if necessary.

### Data synthesis

Our systematic review and meta-analysis will be reported in accordance with the PRISMA guidelines [19, 20]. If two or more studies are available and sufficiently homogeneous in terms of design, population, intervention, comparator and outcome, then we will perform a meta-analysis. Data from RCTs and observational studies will be synthesized separately. For non-randomized studies, we will preferably synthesize data adjusted for any confounders. Adjusted and unadjusted results from non-randomized studies will be pooled and analyzed separately. If significant heterogeneity precludes proceeding to a meta-analysis, then we will perform a systematic narrative synthesis to describe the studies identified.

We note that a number of the selected critical outcomes are conceptually related, including our highest ranked outcome of total/combined duration of respiratory support and invasive mechanical ventilation duration and non-invasive respiratory support duration respectively. Ventilation components are important to separately evaluate due to potential implications for care intensity and care location. We will present our interpretation of critical outcome results which address similar constructs in the discussion section of the planned manuscript reporting our systematic review study findings.

### Statistical analysis

For dichotomous outcomes, we will calculate pooled risk ratios or odds ratios with 95% confidence intervals as appropriate. For continuous outcomes, we will calculate pooled mean differences with 95% confidence intervals. For studies presenting outcome results as median [interquartile range], we will assume a normal distribution and transform these results to mean and standard deviation to enable inclusion in calculation of mean differences [21].

If proceeding to a meta-analysis is appropriate, we plan to perform these analyses using RevMan software (Cochrane Collaboration, Oxford, UK) [22]. We plan to employ a DerSimonian and Laird inverse variance random effects model to calculate pooled effect estimates for each of our prespecified outcomes where data are available. Results will be displayed as Forest plots. We plan to assess statistical heterogeneity between studies using visual inspection of forest plots, the inconsistency index (*I*^2^) and chi-squared testing [23]. If we identify significant heterogeneity, we will investigate potential clinical and methodological reasons for this through subgroup and sensitivity analyses. If 10 or more studies are identified, we will determine the appropriateness of performing a meta-regression analysis to evaluate daily caffeine dose (mg/kg/day) as a potential modifier of treatment effect on outcome [24].

### Subgroup and sensitivity analyses

We plan to evaluate the subgroups listed in Table 2, as we hypothesize that these may be important modifiers of treatment effect. Where subgroup findings are statistically significant, two investigators will independently assess the credibility of these results using the Instrument to assess the Credibility of Effect Modification Analyses (ICEMAN) [25]. We plan to conduct sensitivity analyses comparing studies with high risk of bias versus low risk of bias. We also plan a sensitivity analysis excluding any eligible NICU-based studies with mixed GA, should these be identified.

**Table 2:** Planned subgroup and sensitivity analyses and hypotheses

Subgroup	Hypothesis
Patient age 0–2 years vs > 2 years	We hypothesize improved outcomes in response to caffeine treatment in younger compared with older pediatric patients
Neonates vs non-neonates (neonates are defined as babies with a ^a^Gestational Age (GA) ≤ 44 weeks OR a ^b^Corrected age ≤ 28 days OR as defined by investigators)	We hypothesize improved outcomes in response to caffeine treatment in younger compared with older pediatric patients
Active patient infection, including syndromes caused by an infection (e.g., bronchiolitis, sepsis) vs no active patient infection	We hypothesize improved outcomes in response to caffeine treatment in patients with an infection or infectious syndrome compared to patients with no infection or infectious syndrome
Caffeine loading dose > 20 mg/kg and/or caffeine maintenance dose > 10 mg/kg/day	We hypothesize that higher caffeine dosing may be associated with a stronger treatment effect
Caffeine administration daily (q24 hours) vs more frequent or continuous administration (e.g., q12 hours, q8 hours, q6 hours, or by infusion)	We hypothesize that more frequent or continuous caffeine administration (e.g., q12 hours, q8 hours, q6 hours, or by infusion) will have a stronger treatment effect than once daily dosing regimens
High risk of bias vs low risk of bias	We hypothesize that studies with a high risk of bias may show a stronger treatment effect

a. Gestational age is defined as the gestational age at birth plus days/weeks following birth

b. Corrected age is defined as chronological age (number of days/weeks from date of birth) minus [40 weeks minus gestational age at birth]. From 2 years of age, age is no longer corrected for birth before 40 weeks

### Meta-bias

If 10 or more studies are identified, we will construct a funnel plot to assess for the possibility of publication bias [26].

## Certainty in cumulative evidence

The certainty of the evidence will be reported using the Grading of Recommendations Assessment, Development and Evaluation working group methodology (GRADE) [27]. The GRADE methodology assesses certainty of the aggregate body of evidence considering five domains: risk of bias, imprecision, inconsistency, indirectness, and publication bias. Domain assessments may result in either downgrading or upgrading of evidence, although the latter rarely occurs. We therefore expect to prioritize RCT evidence over evidence from non-randomized studies based on the certainty of evidence assessment. Where meta-analysis is not available and instead available evidence is summarized narratively, we will rate the certainty of evidence using the GRADE approach as suggested by Murad et al. [28]. To evaluate the evidence from included studies, we will create a summary of the findings table using GRADEpro software (www.gradepro.org). Certainty for each outcome will be reported as high, moderate, low, or very low. When data for an outcome are available from multiple study types (e.g., RCT, adjusted non-randomized studies, unadjusted non-randomized studies), we a priori plan to prioritize RCT data in our GRADE Summary of Findings table. To communicate the results of our systematic review, we will employ standardized statements to ensure clarity regarding the estimated effect size and certainty of the evidence for all critical and important outcomes [29].

## Supplementary Information

Supplementary Material 1: Appendix S1. PRISMA-P 2015 Checklist.

Supplementary Material 2: Table S1. OVID Search Strategy.

## Data Availability

Data will be made available upon request to the corresponding author.

## References

[1] Schmidt B, Roberts RS, Davis P, Doyle LW, Barrington KJ, Ohlsson A, et al. Caffeine therapy for apnea of prematurity. N Engl J Med. 2006;354(20):2112–21. 16707748 10.1056/NEJMoa054065

[2] Barrington K, Finer N. The natural history of the appearance of apnea of prematurity. Pediatr Res. 1991;29(4):372–5. 1852531 10.1038/pr.1991.72500

[3] Janvier A, Khairy M, Kokkotis A, Cormier C, Messmer D, Barrington KJ. Apnea is associated with neurodevelopmental impairment in very low birth weight infants. J Perinatol. 2004;24(12):763–8. 15329741 10.1038/sj.jp.7211182

[4] Pergolizzi J, Kraus A, Magnusson P, Breve F, Mitchell K, Raffa R, et al. Treating apnea of prematurity. Cureus. 2022;14(1):e21783. 35251853 10.7759/cureus.21783 PMC8890764

[5] Patrinos ME, Martin RJ. Apnea in the term infant. Semin Fetal Neonatal Med. 2017;22(4):240–4. 28438477 10.1016/j.siny.2017.04.003

[6] Pappano DA. Respiratory Virus-Associated Apnea. Clinician.com. 12(1):1–12.

[7] Tieder JS, Bonkowsky JL, Etzel RA, Franklin WH, Gremse DA, Herman B, et al. Brief resolved unexplained events (formerly apparent life-threatening events) and evaluation of lower-risk infants. Pediatrics. 2016;137(5):e20160590. 27244835 10.1542/peds.2016-0590

[8] Ochi M, Nosaka N, Knaup E, Tsukahara K, Kikkawa T, Fujii Y, et al. Recurrent apnea in an infant with pertussis due to household transmission. Clin Case Rep. 2017;5(3):241–5. 28265381 10.1002/ccr3.765 PMC5331238

[9] Kilgore PE, Salim AM, Zervos MJ, Schmitt HJ. Pertussis: Microbiology, Disease, Treatment, and Prevention. Clin Microbiol Rev. 2016;29(3):449–86. 27029594 10.1128/CMR.00083-15 PMC4861987

[10] Heuzé N, Goyer I, Porcheret F, Denis M, Faucon C, Jokic M, et al. Caffeine treatment for bronchiolitis-related apnea in the pediatric intensive care unit. Arch Pediatr. 2020;27(1):18–23. 31776076 10.1016/j.arcped.2019.10.009

[11] Principi T, Fraser DD, Morrison GC, Farsi SA, Carrelas JF, Maurice EA, et al. Complications of mechanical ventilation in the pediatric population. Pediatr Pulmonol. 2011;46(5):452–7. 21194139 10.1002/ppul.21389

[12] Manning JC, Pinto NP, Rennick JE, Colville G, Curley MAQ. Conceptualizing Post Intensive Care Syndrome in Children-The PICS-p framework. Pediatr Crit Care Med. 2018;19(4):298–300. 29406379 10.1097/PCC.0000000000001476

[13] Moher D, Shamseer L, Clarke M, Ghersi D, Liberati A, Petticrew M, et al. Preferred reporting items for systematic review and meta-analysis protocols (PRISMA-P) 2015 statement. Syst Rev. 2015;4(1):1. 25554246 10.1186/2046-4053-4-1 PMC4320440

[14] Shamseer L, Moher D, Clarke M, Ghersi D, Liberati A, Petticrew M, et al. Preferred reporting items for systematic review and meta-analysis protocols (PRISMA-P) 2015: elaboration and explanation. BMJ. 2015;2(349):g7647. 10.1136/bmj.g7647 25555855

[15] Cuello-Garcia CA, Santesso N, Morgan RL, Verbeek J, Thayer K, Ansari MT, et al. GRADE guidance 24 optimizing the integration of randomized and non-randomized studies of interventions in evidence syntheses and health guidelines. J Clin Epidemiol. 2022;1(142):200–8. 10.1016/j.jclinepi.2021.11.026 PMC8982640 34800676

[16] Schlapbach LJ, Ramnarayan P, Gibbons KS, Morrow BM, Napolitano N, Tume LN, et al. Building global collaborative research networks in paediatric critical care: a roadmap. Lancet Child Adolesc Health. 2025;9(2):138–50. 39718171 10.1016/S2352-4642(24)00303-1

[17] Sterne JAC, Savović J, Page MJ, Elbers RG, Blencowe NS, Boutron I, et al. RoB 2: a revised tool for assessing risk of bias in randomised trials. BMJ. 2019;28(366):l4898. 10.1136/bmj.l4898 31462531

[18] Risk of bias tools - ROBINS-I V2 tool. Available from: https://www.riskofbias.info/welcome/robins-i-v2. Cited 2025 Sep 18.

[19] Page MJ, McKenzie JE, Bossuyt PM, Boutron I, Hoffmann TC, Mulrow CD, et al. The PRISMA 2020 statement: an updated guideline for reporting systematic reviews. BMJ. 2021;29(372):n71. 10.1136/bmj.n71 PMC8005924 33782057

[20] Page MJ, Moher D, Bossuyt PM, Boutron I, Hoffmann TC, Mulrow CD, et al. PRISMA 2020 explanation and elaboration: updated guidance and exemplars for reporting systematic reviews. BMJ. 2021;29(372):n160. 10.1136/bmj.n160 PMC8005925 33781993

[21] Wan X, Wang W, Liu J, Tong T. Estimating the sample mean and standard deviation from the sample size, median, range and/or interquartile range. BMC Med Res Methodol. 2014;14(1):135. 25524443 10.1186/1471-2288-14-135 PMC4383202

[22] The Cochrane Collaboration. Review Manager (RevMan). 2024. Available from: revman.cochrane.org

[23] Higgins J, Thomas J, Chandler J, Cumpston M, Li T, Page M, et al editors. Cochrane handbook for systematic reviews of interventions. version 6.5. Cochrane; 2024.

[24] Higgins J, Thomas J, Chandler J, Cumpston M, Li T, Page M, editors. Chapter 10: Analysing data and undertaking meta-analyses. In: Cochrane Handbook for Systematic Reviews of Interventions. version 6.5. Available from: http://www.cochrane.org/handbook. Cited 2026 Feb 13.

[25] Schandelmaier S, Briel M, Varadhan R, Schmid CH, Devasenapathy N, Hayward RA, et al. Development of the Instrument to assess the Credibility of Effect Modification Analyses (ICEMAN) in randomized controlled trials and meta-analyses. CMAJ. 2020;192(32):E901–6. 32778601 10.1503/cmaj.200077 PMC7829020

[26] Egger M, Smith GD, Schneider M, Minder C. Bias in meta-analysis detected by a simple, graphical test. BMJ. 1997;315(7109):629–34. 9310563 10.1136/bmj.315.7109.629 PMC2127453

[27] Guyatt GH, Oxman AD, Vist GE, Kunz R, Falck-Ytter Y, Alonso-Coello P, et al. GRADE: an emerging consensus on rating quality of evidence and strength of recommendations. BMJ. 2008;336(7650):924–6. 18436948 10.1136/bmj.39489.470347.AD PMC2335261

[28] Murad MH, Mustafa RA, Schünemann HJ, Sultan S, Santesso N. Rating the certainty in evidence in the absence of a single estimate of effect. Evid Based Med. 2017;22(3):85–7. 28320705 10.1136/ebmed-2017-110668 PMC5502230

[29] Santesso N, Glenton C, Dahm P, Garner P, Akl EA, Alper B, et al. GRADE guidelines 26: informative statements to communicate the findings of systematic reviews of interventions. J Clin Epidemiol. 2020;1(119):126–35. 10.1016/j.jclinepi.2019.10.014 31711912

