# Supplementary material for: Caffeine treatment for apnea in infants and children: protocol for a systematic review and meta-analysis
Source: Syst Rev. 2026 Jun 8;15:221. doi: 10.1186/s13643-026-03222-w (PMC13471563; doi:10.1186/s13643-026-03222-w)
Supplement: Supplementary file 2 [file 13643_2026_3222_MOESM2_ESM.docx]

**Table S1.** OVID Search Strategy

*OVID*

Database(s): **Embase** 1974 to 2026 February 13**, Ovid MEDLINE(R) ALL** 1946 to February 16, 2026**, EBM Reviews - Cochrane Central Register of Controlled Trials** January 2026
Search Strategy;

| **Line #** | **Search Terms** |
| --- | --- |
| 1 | (exp Critical Care/ or Critical Care Outcomes/ or Critical Care Nursing/ or Critical Illness/ or exp Intensive Care Units, Pediatric/) use medall,cctr |
| 2 | (*Critical Care Outcome/ or *Critical Illness/ or *Critically Ill Patient/ or exp *Intensive Care/ or *Pediatric Intensive Care Unit/ or *Neonatal Intensive Care Unit/ or *Pediatric Ward/) use oemezd |
| 3 | (((critical* or emergenc* or intensive* or lifethreaten* or life-threaten* or neurointensive* or neuro-intensive* or neurocritical* or neuro-critical* or stepdown or step down) adj (care or department? or healthcare or health-care or ill or illness* or room? or therap* or treatment* or unit? or ward?)) or comatose or ("in" adj2 coma)).tw,kf,kw,jn. |
| 4 | (((acute* or critical* or lifethreaten* or life-threaten* or serious* or sever*) adj2 (disease? or injur* or patient? or sick* or unwell or wound*)) or (high* adj2 (acuity or care))).ti,kf,kw. or (((acute* or critical* or lifethreaten* or life-threaten* or serious* or sever*) adj2 (disease? or injur* or patient? or sick* or unwell or wound*)) or (high* adj2 (acuity or care))).ab. /freq=2 |
| 5 | ((adolescen* or babies or baby or child* or infant? or juvenile* or neonat* or neo-nat* or newborn* or new-born* or p?ediatric* or perinat* or peri-nat* or youth*) adj3 (ICU or ICUs or department? or unit? or ward?)).tw,kf,kw. |
| 6 | (NICU or NICUs or PICU or PICUs).tw,kf,kw. |
| 7 | (burn unit? or burn ward? or cardiac care unit? or cardiac care ward? or coronary care unit? or coronary care ward? or high dependency unit? or high dependency ward? or intensive care unit? or intensive care ward? or intensive therapy unit? or intensive therapy ward? or recovery room? or recovery ward? or respiratory care unit? or respiratory care ward?).ti,kf,kw. or (burn unit? or burn ward? or cardiac care unit? or cardiac care ward? or coronary care unit? or coronary care ward? or high dependency unit? or high dependency ward? or intensive care unit? or intensive care ward? or intensive therapy unit? or intensive therapy ward? or recovery room? or recovery ward? or respiratory care unit? or respiratory care ward?).ab. /freq=2 |
| 8 | (Advanced Trauma Life Support Care/ or Emergencies/ or Emergency Medical Services/ or Emergency Medicine/ or Emergency Nursing/ or Emergency Room Visits/ or exp Emergency Service, Hospital/ or Emergency Treatment/ or Evidence-based Emergency Medicine/ or Pediatric Emergency Medicine/) use medall,cctr |
| 9 | (*Advanced Trauma Life Support/ or *Emergency/ or exp *Emergency Care/ or exp *Emergency Health Service/ or *Emergency Medicine/ or *Emergency Nursing/ or exp *Emergency Visit/ or *Emergency Treatment/ or *Evidence-based Emergency Medicine/ or Pediatric Emergency Medicine/) use oemezd |
| 10 | ((("A and E" or "A-E" or "AE" or "ED" or "ER" or emergencies or emergency) adj2 (department* or hospital* or medicine or room? or service? or unit? or ward?)) or trauma centre? or trauma center? or trauma unit? or trauma ward?).tw,kf,kw,jn. |
| 11 | (Adolescent, Hospitalized/ or Child, Hospitalized/ or Hospitalization/ or Inpatients/) use medall,cctr |
| 12 | (Child Hospitalization/ or Hospitalization/ or Hospitalized Adolescent/ or Hospitalized Child/ or Hospitalized Infant/ or Hospital Patient/) use oemezd |
| 13 | (hospital* or inpatient* or in-patient*).tw,kf,kw. |
| 14 | or/1-13 |
| 15 | exp Adolescent/ or exp Child/ or exp Infant/ or exp Juvenile/ or Pediatric Patient/ or exp Pediatrics/ or exp Pediatricians/ or Pediatric Anesthesia/ or (adolescen* or babies or baby or boy or boyhood or boys or child* or girl or girlhood or girls or infan* or juvenil* or kid or kids or minor? or neonat* or neo-nat* or newborn* or new-born* or p?ediatric* or perinat* or peri-nat* or schoolage* or school-age* or schoolchild* or school-child* or teen* or toddler? or underage* or under-age* or youth*).mp,hw,ia,in,jw. |
| 16 | 14 and 15 [Critically Ill Children (Population)] |
| 17 | exp Adult/ not (exp Adult/ and (exp Child/ or exp Adolescent/ or exp Infant/ or exp Juvenile/)) [Adult Only] |
| 18 | (exp animals/ or exp animal experimentation/ or exp animal experiment/ or exp models animal/ or nonhuman/ or exp vertebrate/ or exp vertebrates/) not (exp humans/ or exp human experimentation/ or exp human experiment/) [Animal Only] |
| 19 | 16 not (17 or 18) [Critically Ill Children, Adult/Animal Only records removed] |
| 20 | Caffeine/ [all] |
| 21 | Caffeine Citrate.nm. use medall,cctr |
| 22 | Caffeine Citrate/ use oemezd |
| 23 | (caffein* or "1,3,7 trimethyl 2,6 dioxopurine" or "1,3,7-trimethylxanthine" or animine or cafeine or caffedrine or caffein or coffein or coffeine or "coffeinum n" or "coffeinum purrum" or dexitac or durvitan or guaranine or guarin or methyltheobromine or "percoffedrinol n" or percutafeine or thein or trimethylxanthine or vivarin or "58-08-2").tw,kf,kw. |
| 24 | (caffeine citrate* or blectifor or cafcit or cafnea or citrated caffeine or fenapin or gencebok or "npc 11" or npc11 or nymusa or peyona or respia or "69−22−7").tw,kf,kw. |
| 25 | or/20-24 [Caffeine/Caffeine Citrate] |
| 26 | Apnea/ [all] |
| 27 | apn?e*.tw,kf,kw. |
| 28 | or/26-27 [Apnea] |
| 29 | 19 and 25 and 28 [Final results, all databases, Critically Ill Children + Caffeine + Apnea, Adult/Animal Only records removed] |
